# Integrated bioinformatics analysis and experimental validation reveals fatty acid metabolism-related prognostic signature and immune responses for uterine corpus endometrial carcinoma

**DOI:** 10.3389/fonc.2022.1030246

**Published:** 2022-11-09

**Authors:** Chenrui Guo, Yan He, Leiming Chen, Yuan Li, Yajun Wang, Yunlei Bao, Ni Zeng, Feng Jiang, Hang Zhou, Le Zhang

**Affiliations:** ^1^ Department of Abdominal Oncology, The Second Affiliated Hospital of Zunyi Medical University, Zunyi, China; ^2^ Department of Gynecology, Shidong Hospital, Shidong Hospital Affiliated to University of Shanghai For Science and Technology, Shanghai, China; ^3^ Department of Laboratory Medicine, Obstetrics and Gynecology Hospital of Fudan University, Shanghai, China; ^4^ Department of Dermatology, The Fifth People’s Hospital of Hainan Province, Haikou, China; ^5^ Department of Neonatology, Obstetrics and Gynecology Hospital of Fudan University, Shanghai, China; ^6^ Department of Dermatology, Affiliated Hospital of Zunyi Medical University, Zunyi, China; ^7^ Department of Anesthesiology, Obstetrics and Gynecology Hospital of Fudan University, Shanghai, China

**Keywords:** uterine corpus endometrial carcinoma, fatty acid metabolism, risk signature, immune status, prognosis

## Abstract

**Background:**

Uterine corpus endometrial carcinoma (UCEC) is the third most common gynecologic malignancy. Fatty acid metabolism (FAM) is an essential metabolic process in the immune microenvironment that occurs reprogramming in the presence of tumor signaling and nutrient competition. This study aimed to identify the fatty acid metabolism-related genes (FAMGs) to develop a risk signature for predicting UCEC.

**Methods:**

The differentially expressed FAMGs between UCEC samples and controls from TCGA database were discovered. A prognostic signature was then constructed by univariate, least absolute shrinkage and selection operator (LASSO) and multivariate Cox regression analyses. Based on the median risk score, UCEC samples were categorized into high- and low-FAMGs groups. Kaplan-Meier (K-M) curve was applied to determine patients’ overall survival (OS). The independent prognostic value was assessed by uni- and multivariate analyses. The associations between the risk score and immune status, immune score, and drug resistance were evaluated. Quantitative Real-time PCR (qRT-PCR) was utilized to confirm FAMGs expression levels in UCEC cells.

**Results:**

We built a 10-FAMGs prognostic signature and examined the gene mutation and copy number variations (CNV). Patients with a high-FAMGs had a worse prognosis compared to low-FAMGs patients in TCGA train and test sets. We demonstrated that FAMGs-based risk signature was a significant independent prognostic predictor of UCEC. A nomogram was also created incorporating this risk model and clinicopathological features, with high prognostic performance for UCEC. The immune status of each group was varied, and immune score was higher in a low-FAMGs group. HLA-related genes such as DRB1, DMA, DMB, and DQB2 had higher expression levels in the low-FAMGs group. Meanwhile, high-FAMGs patients were likely to response more strongly to the targeted drugs Bortezomib, Foretinib and Gefitinib. The qRT-PCR evidence further verified the significant expression of FAMGs in this signature.

**Conclusions:**

A FAMGs-based risk signature might be considered as an independent prognostic indicator to predict UCEC prognosis, evaluate immune status and provide a new direction for therapeutic strategies.

## Introduction

Uterine corpus endometrial carcinoma (UCEC), a frequent gynecological malignant tumor, has been rapidly increasing in recent years. According to the 2022 cancer statistics, there were an estimated 65,950 cases and 12,550 deaths from uterine corpus cancer in United State (1). In China, it is estimated that the number of new cases was 84,520, and deaths were 17,543 of UCEC in young women (2). It, therefore, remains a major public health issue around the world. Despite surgery, chemotherapy, radiotherapy and brachytherapy currently employed to UCEC intervention, there are still considerable numbers of women with more aggressive lesions whose prognosis is dismal (3). A reliable prognosis assessment is the foundation of effective therapy. However, the present predictive system based on clinical, pathological, imaging, and biological features are insufficient to interpret the progressive and prognostic heterogeneity of UCEC (4). Consequently, exploring effective biomarkers to identify individuals with a high risk of recurrence is helpful for precise therapy.

Tumor growth is deeply reliant on the tumor microenvironment (TME), which is characterized by hypoxia, acidity, and nutrition deprivation because tumor cell proliferation is faster than angiogenesis (5). Consequently, tumor cells displayed distinctive metabolic properties from normal cells to handle a variety of adverse situations *via* a metabolic reprogramming process that supports their growth and survival once the carcinogenic signal is blocked (6). lipid metabolism is one of three primary energy metabolisms of cells, of which fatty acid metabolism (FAM) is a critical metabolic pathway and plays an important role in cancer pathophysiology (7). Specifically, the fatty acid in particular aids cancer cells in not only maintaining membrane biosynthesis but also supplying a major energy source during metabolic pressure. The FAM pattern tends to be different among various cells and tissues. Previous studies have shown that certain expression patterns of fatty acid metabolism-related genes (FAMGs) were connected with proliferation, prognosis, and immunity of glioma, colorectal cancer, or breast cancer (8–11). However, the pattern of FAMGs in UCEC has not been explored.

As bioinformatic technology develops, numerous approaches have been employed to define meaningful biomarkers (12, 13). In this study, the potential value of FAM in UCEC samples obtained from TCGA database was evaluated by using bioinformatics. We analyzed the differential expression of FAMGs, and selected genes strongly correlated with UCEC prognosis to develop a FAMGs-based risk signature. A scoring system was created to evaluate the probability of survival, as well as immune status and drug sensitivity of UCEC. This risk model offered a novel insight on UCEC prognosis and therapeutic options.

## Materials and methods

### Data sources

A total of 539 UCEC and 35 normal endometrial cases were retrieved from TCGA database (https://portal.gdc.cancer.gov/). Patients were allocated to TCGA training or test cohort in a 1:1 ratio at random. FAM gene sets, including *KEGG FAM pathways, Hallmark FAM genes, and Reactome FAM genes*, were acquired from the MSigDB v7.4. A total of 309 FAMGs were finally ascertained after removing the overlapping genes (14).

### Construction and verification of a prognostic signature

The DEGs linked to FAM between UCEC, and normal tissues were screened by R “limma” package (15), with |logFC|>0 and FDR-adjusted *P<*0.05. Upon these FAM-DEGs, the overall survival (OS) related genes with *P*< 0.05 were selected by using a univariate cox analysis. Then, candidate FAMGs were confirmed by using the least absolute shrinkage and selection operator (LASSO) and multivariate cox regression analysis *via* R “glmnet” packages (16).

The risk score is computed by using this formula: $risk\;score=\sum_{i=0}^{n}\left(coefi\times Genei\right)$ , where *Gene_i_* denotes the expression level of gene *i* and *coef_i_* denote the regression coefficient of gene *i*. Based on median risk score, UCEC patients were divided into a low- or high-FAMGs group in TCGA training or test set. The log-rank test was utilized to compare the difference in survival status between two groups *via* R “rms” package. Kaplan-Meier (K-M) analysis of OS or PFS was performed *via* R “survival” package. To reflect the predictive power of risk model, we plotted the time-dependent receiver operating characteristic (ROC) curve and area under the curve (AUC) for 1-year, 3-year, and 5-year OS *via* R “timeROC” package (17).

Gene Ontology (GO) (18) and Kyoto Encyclopedia of Genes and Genomes (KEGG) (19) functional enrichment analysis of FAMGs in UCEC were conducted by R “ClusterProfiler” package, where *P*< 0.05 indicates a statistical difference.

### Establishment of a FAMGs-related nomogram

To verify the independence of the FAMGs‐based risk signature, we ran uni- and multivariate cox analyses on risk score and other clinical factors. Then, using the above variables, we established a FAMGs-related clinicopathologic nomogram *via* R “rms”, “nomogramEx”, and “regplot” packages (20). Then, ROC and calibration curves were utilized to examine the accuracy and discrimination of the nomogram.

### Assessment of immune status and drug sensitivity

The association between tumor immune microenvironment (TIME) and this prognostic signature was further assessed. The single-sample gene-set enrichment analysis (ssGSEA) was utilized to quantify the immune activity in high- and low-FAMGs groups. Tumor Immune Dysfunction and Exclusion (TIDE) algorithm was conducted to evaluate the tumor immune escape in two groups based on FAMGs. ESTIMATE algorithm was used to determine the TME score *via* R software. The expressions of HLA-genes between high- and low-FAMGs group were further compared. To investigate differences in therapeutic effects of small-molecule drugs between two groups, we calculated the half-maximal inhibitory concentration (IC50) values of drugs commonly for UCEC treatment *via* R “pRRophetic” package [21fron].

### Cell culture and qRT-PCR

Human endometriosis cell line hEM15A and UCEC cell ANC3A were cultured in DMEM with 10% FBS in a 5% CO2 incubator at 37°C. Total RNA was extracted by utilizing Trizol reagent (Invitrogen, USA), then cDNAs were generated with a HiScript Synthesis kit (Vazyme, China). Quantitative real-time PCR (qRT-PCR) was completed by utilizing the Fast SYBR Green Master Mix (Roche, USA) on a StepOnePlus Real-Time PCR system (Applied Biosystems, USA). Primer sequences in our work are described in **Table S1**.

## Results

### Landscape of FAMGs expression in UCEC

**Figure 1:**
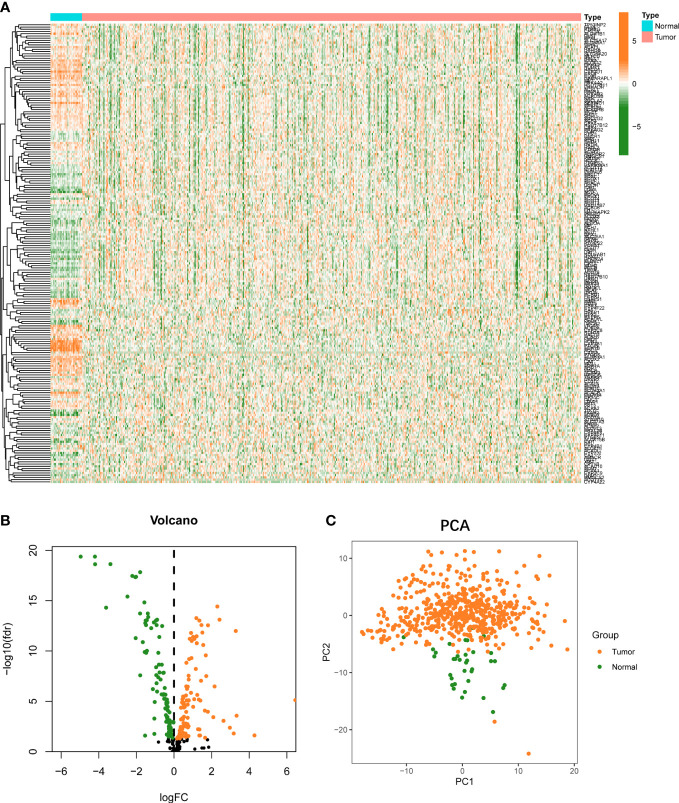
Landscape of FAMGs expressions in UCEC and normal endometrial tissues. **(A)** Heatmap for 309 FAMGs in TCGA cohort. **(B)** Volcano plot for different expressions of FAMGs. **(C)** PCA analysis for FAMGs to distinguish tumors (n = 554) from normal samples (n = 35). FAMGs fatty acid metabolism-related genes. UCEC: Uterine corpus endometrial carcinoma. PCA: principal component analysis.

There are 554 UCEC and 35 normal samples retrieved from TCGA dataset. Differentially expressed FAMGs between tumor and normal were presented in a heat map (**Figure 1A**). The volcanic diagram displayed 100 up-regulated FAMGs and 106 down-regulated FAMGs (**Figure 1B**). Principal component analysis (PCA) was applied to evaluate sample heterogeneity, and the results showed a significant difference (**Figure 1C**). We also included more normal samples from GTEx database (n=78) to verify this difference (**Figure S1**). 131 up-regulated FAMGs and 103 down-regulated FAMGs were found. Most of the different FAMGs overlapped. These suggested that FAMGs might have a potential ability to differentiate normal patients from UCEC.

### Identification of prognostic FAMGs and genomic variance analysis

**Figure 2:**
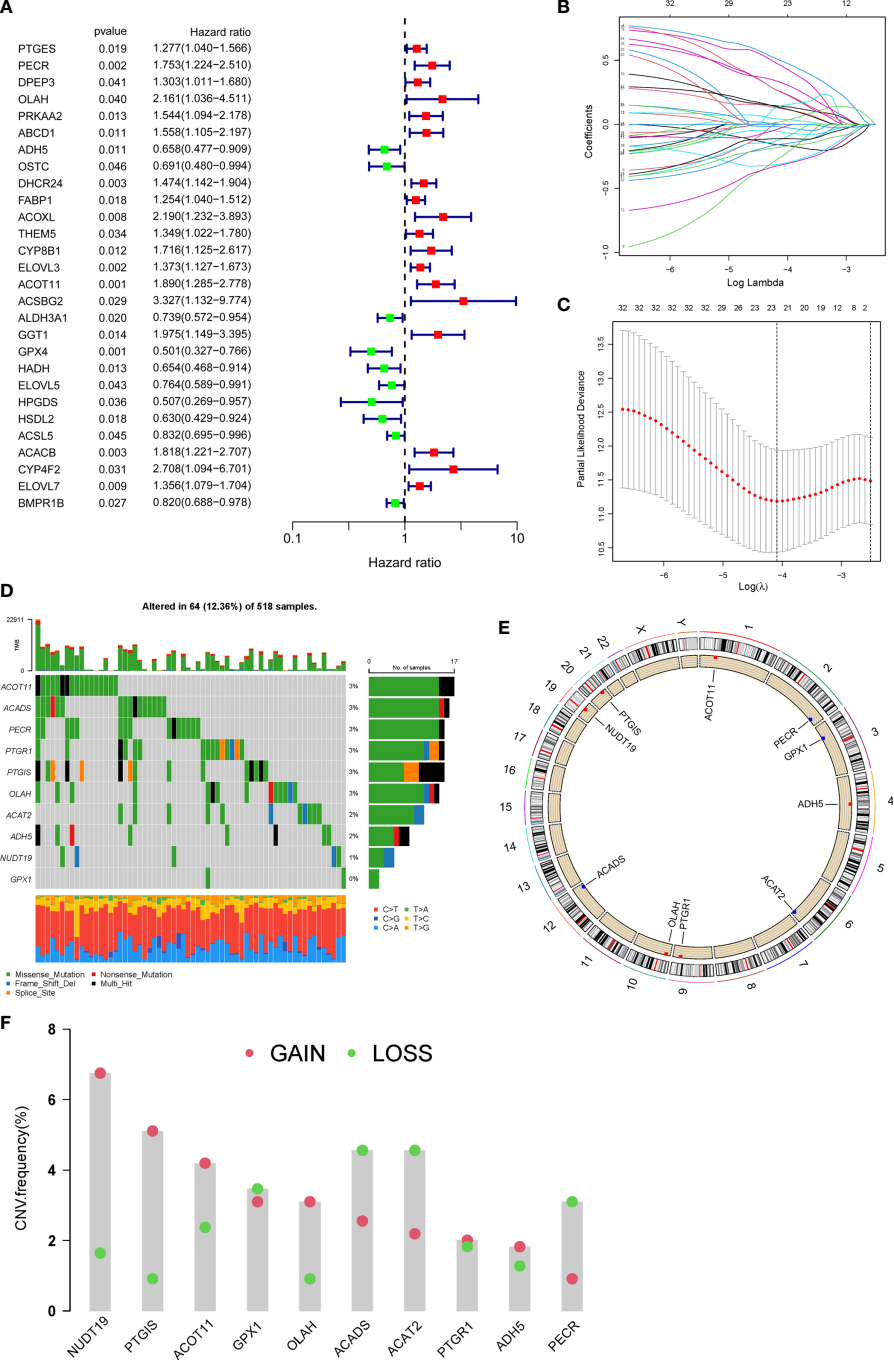
Identification of prognostic FAMGs and their characteristic analysis. **(A)** Forrest plot of 28 FAMGs related with prognosis by univariate regression analysis. **(B)** LASSO regression analysis. **(C)** Partial likelihood deviance for LASSO regression. **(D)** Profiles of genetic mutation in UCEC patients. **(E)** CNV alteration of FAMGs on chromosomes. **(F)** Frequencies of CNV gain, loss, and non-CNV among FAMGs. FAMGs: fatty acid metabolism-related genes. LASSO: least absolute shrinkage and selection operator. UCEC: Uterine corpus endometrial carcinoma. CNV: copy number variation. .

Firstly, 206 differentially expressed FAMGs were subjected to univariate Cox analysis, and 28 prognosis-related genes were identified with a *P*< 0.05 (**Figure 2A**). Next, LASSO and multiple Cox analyses were utilized to shrink the range of FAMGs. At last, 10 FAMGs including upregulated PECR, OLAH, ACOT11, ACAT2, NUDT19, PTGIS, and downregulated GPX1, ADH5, PTGR1, ACADS were identified for prognostic risk model (**Figures 2B, C**).

The incidence of somatic mutation of FAMGs in UCEC patients was calculated from TCGA cohort. There are 64/518 (12.36%) UCEC patients who experienced mutations of these 10 FAMGs, with a frequency from 0 to 3% (**Figure 2D**). Among them, ACOT11, ACADS, PECR, PTGR1, PTGIS, OLAH had 3% mutation frequency, followed by ACAT2, ADH5, NUDT19, while GPX1 did not have any mutations. Next, we investigated the CNV of FAMGs and found its prevalence in these 10 FAMGs. The location of CNV changes of the FAMGs on each of their respective chromosomes was shown in **Figure 2E**. Among them, NUDT19, PTGIS, ACOT11, OLAH, PTGR1, and ADH5 exhibited widespread CNV gain, while GPX1, ACADS ACAT2, and PECR had CNV loss (**Figure 2F**).

### Construction and validation of FAMGs-based risk signature

**Figure 3:**
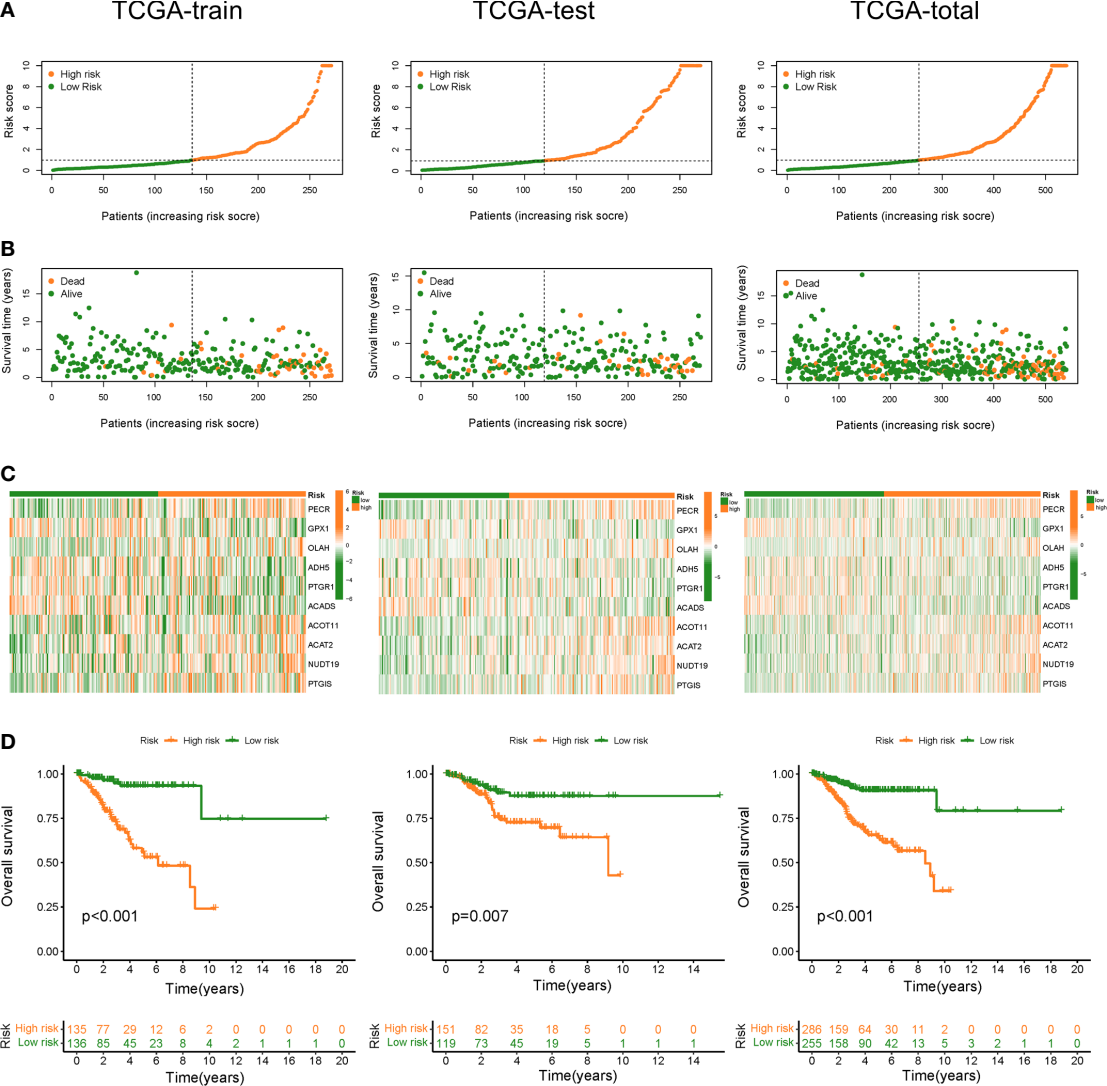
Construction and validation of FAMGs-based risk signature in TCGA train set, test set and total set. **(A)** Distribution of risk score in high- and low- FAMGs groups. **(B)** Dot pot of survival status with increasing risk score. **(C)** Heat map of FAMGs expressions in two groups. **(D)** K-M curve for patients’ OS. FAMGs: fatty acid metabolism-related genes. K-M: Kaplan-Meier. OS: overall survival.

Based on FAMGs median risk score, UCEC patients were categorized into high- and low-FAMGs groups in TCGA training cohort, test cohort and total cohort (**Figures 3A**). The proportion of alive patients in high-FAMGs group was less than that of low-FAMGs patients among these datasets (**Figure 3B**). The distribution of these 10 FAMGs expression levels UCEC patients was shown in a heatmap (**Figure 3C**). K-M curve revealed that low-FAMGs patients’ OS was considerably longer than high-FAMGs patients among these cohorts (**Figure 3D**), as did their PFS time (**Figure S2**).

### Functional annotation

Further, we identified 835 differentially expressed genes between high- and low-FAMGs groups. To examine the possible biological properties in UCEC, we conducted a functional enrichment analysis of these genes. GO analysis showed that signaling receptor activator activity, tubulin binding, tubulin binding and fatty acid synthase activity were significantly enriched in biological processes (**Figure S3A**). KEGG revealed the enrichment in microtubule-based movement, cilium assembly, cilium movement and pattern specification process (**Figure S3B**).

### Independent prognostic value of FAMGs-based risk signature

**Figure 4:**
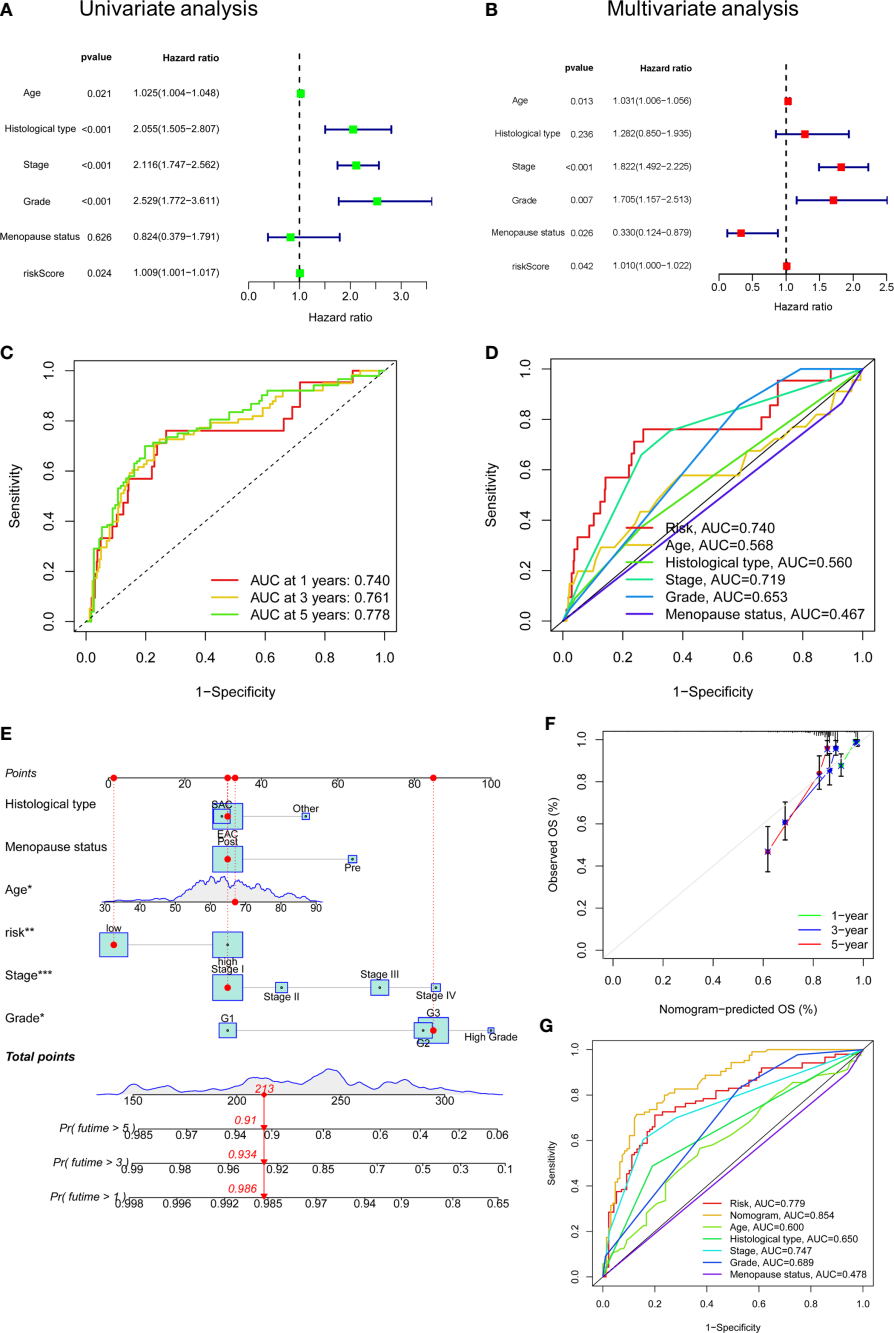
Independent prognostic value of risk score and a nomogram construction. **(A)** Univariate Cox analysis of risk score and clinicopathologic parameters. **(B)** Multivariate Cox analysis. **(C)** ROC curve of risk score for 1-, 3- and 5- years’ OS. **(D)** ROC curves of risk score, age, histological type, stage, grade, menopause status. **(E)** A Nomogram consisting of risk score, age, grade, stage, histological type, and menopause status. **(F)** Calibration curves for patients’ OS at 1, 2, and 3 years. **(G)** ROC curves for the nomogram and clinicopathologic factors. ROC: receiver operating characteristic. OS: overall survival.

To elucidate whether FAMGs-based risk signature is an independent prognostic indicator for UCEC, uni- and multivariate Cox analyses were carried out in TCGA total set. As shown in **Figure 4A**, age (*P* = 0.021), histological type (*P*< 0.001), stage (*P*< 0.001), grade (*P*< 0.001), and risk score (*P* = 0.024) had significant correlation with OS. Multivariate Cox analysis (**Figure 4B**) showed that age (*P* = 0.013), stage (*P*< 0.001), grade (*P* = 0.007) and menopause status (*P* = 0.026) and risk score (*P* = 0.042) were related to OS. We then performed a time-dependent ROC curve to test this risk model’s predicting ability and accuracy (**Figure 4C**). The result showed that AUC of the 1‐, 3‐, or 5‐year OS was 0.740, 0.761, 0.778, respectively. Importantly, the AUC value for this risk model was 0.740, which was higher than that for the age (0.568), histological type (0.560), and stage (0.719) (**Figure 4D**). These results indicated that our FAMGs-based risk signature exhibited a great independent predictive value in UCEC.

### Establishment of FAMGs-related nomogram

A nomogram integrating the risk model, age, grade, stage, histological type, and menopause status was created to predict UCEC patients’ OS (**Figure 4E**). By summing the point for each prognostic factor, a total point was generated for each patient, and higher total points meant a worse outcome. The calibration plot showed a close agreement with the ideal model, demonstrating the nomogram’s perfect stability and discrimination (**Figure 4F**). ROC curve showed that this nomogram (AUC = 0.854) had a superior predictive ability than a separate parameter, such as age (AUC = 0.600), stage (AUC = 0.747), or risk model (AUC = 0.779) (**Figure 4G**).

### Prognostic power of FAMGs in UCEC patients

**Figure 5:**
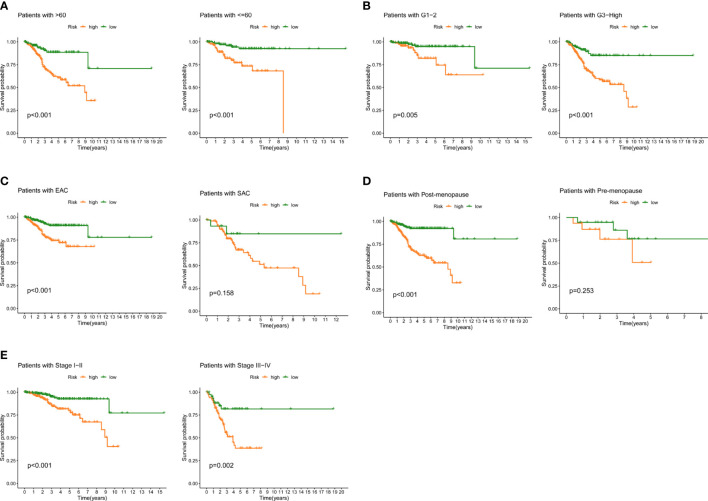
Prognostic power in different clinical subgroups. K-M curve analyses for patient subgroups, including **(A)** age, **(B)** grade, **(C)** histological type, **(D)** menopause, **(E)** stage. K-M: Kaplan-Meier.

We examined the prognostic power of FAMGs-based risk signature for UCEC patients under different clinicopathological factors, including age, grade, histological type, menopause status and stage. K-M analysis for each subgroup revealed that low-FAMGs patients have longer OS than high-FAMGs patients, no matter their age, grade, and stage, as is the same situation in patients with EAC or post-menopause (**Figure 5**). These suggested that FAMGs-based risk signature has strong predictive power in most populations with different clinical features.

### Analysis of immune microenvironment

**Figure 6:**
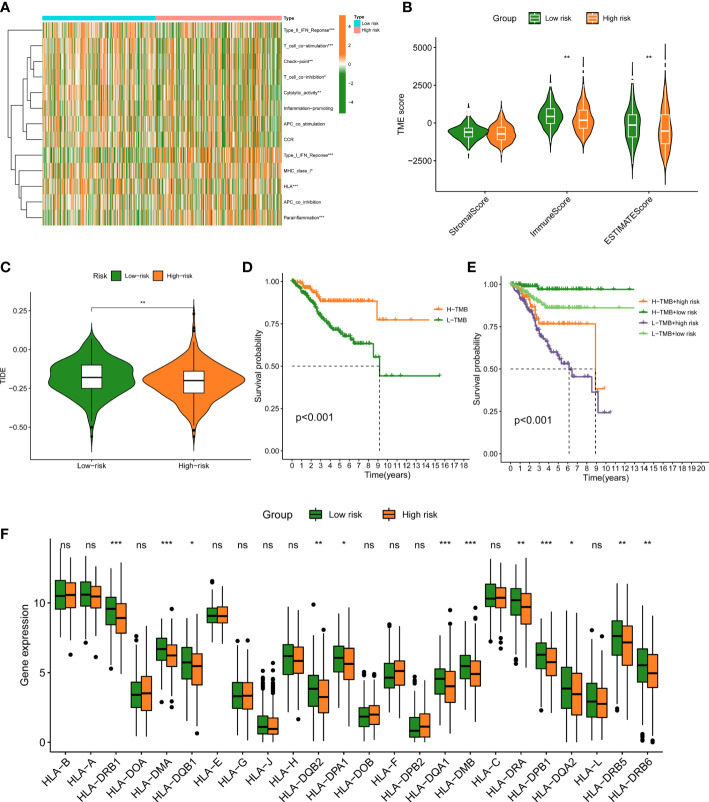
Immune landscape between low- and high-FAMGs group. **(A)** Heat map of immune status between two groups. **(B)** TME Score in different groups. **(C)** TIDE score in different groups. **(D)** K-M analysis of high- and low-TMB group. **(E)** K-M analysis of four groups classified by TMB and FAMGs risk score. **(F)** Box plot of HLA-related gene expressions in two groups. ^∗^ *P*<0.05, ^∗∗^ *P*<0.01, and ^∗∗∗^ *P*<0.001. FAMGs: fatty acid metabolism-related genes. TME: Tumor microenvironment. TIDE: Tumor Immune Dysfunction and Exclusion. K-M: Kaplan-Meier. TMB: tumor mutational burden. ns, not significant.

We further evaluate the relationship between TIME and this prognostic signature. Immune status of low- and high-FAMGs patients revealed some degree of heterogeneity (**Figure 6A**). In addition, high-FAMGs group had a lower TIDE score compared with low-FAMGs group (**Figure 6B**). Regarding the TME score, high-FAMGs patients had lower immune scores, and ESTIMATE score than low-FAMGs patients, but no significant difference in Stromal score between two groups (**Figure 6C**). Furthermore, we observed that high-TMB (tumor mutational burden) was linked to a better OS (*P*< 0.001, **Figure 6D**). We then combined FAMGs with TMB to divide patients into high-TMB/low-risk, low-TMB/low-risk, high-TMB/high-risk, and low-TMB/high-risk groups. As seen in **Figure 6E**, a significant difference among all groups was identified (*P*< 0.001), with high-TMB/low-risk group showing the highest OS. Furthermore, we also found that most of HLA-related genes, such as DRB1, DMA, DQB2, DMB were expressed significantly higher in low-FAMGs group (**Figure 6F**).

### Response to therapeutic drugs

**Figure 7:**
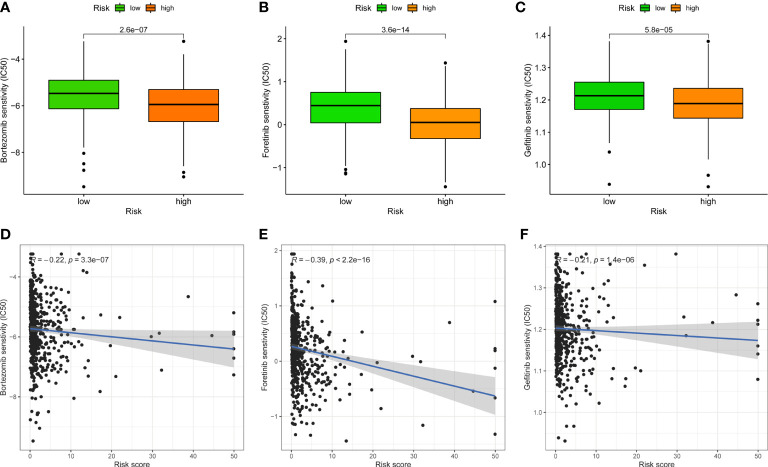
Effect of FAMGs-based risk signature in targeted UCEC therapy. **(A–C)** Comparation of therapeutic sensitivity between low- and high-FAMGs groups. **(D–F)** Relationship of risk score and estimated IC50 value. FAMGs: fatty acid metabolism-related genes. UCEC: Uterine corpus endometrial carcinoma. IC50: half-maximal inhibitory concentration.

Given that the risk score is linked to a poor prognosis, it is necessary to investigate the impact of FAMGs-based risk signature on the drug resistance for UCEC. IC50 was designed to predict the therapeutic response to common targeted drugs. As shown in **Figures 7A-C**, low-FAMGs samples had greater IC50 values of Bortezomib, Foretinib and Gefitinib compared with high-FAMGs patients. Moreover, the risk score is inversely correlated with drug sensitivity (**Figures 7D-F**). These results suggested that high-FAMGs patients were more responsive to Bortezomib, Foretinib and Gefitinib.

### Validation of FAMGs expression

**Figure 8:**
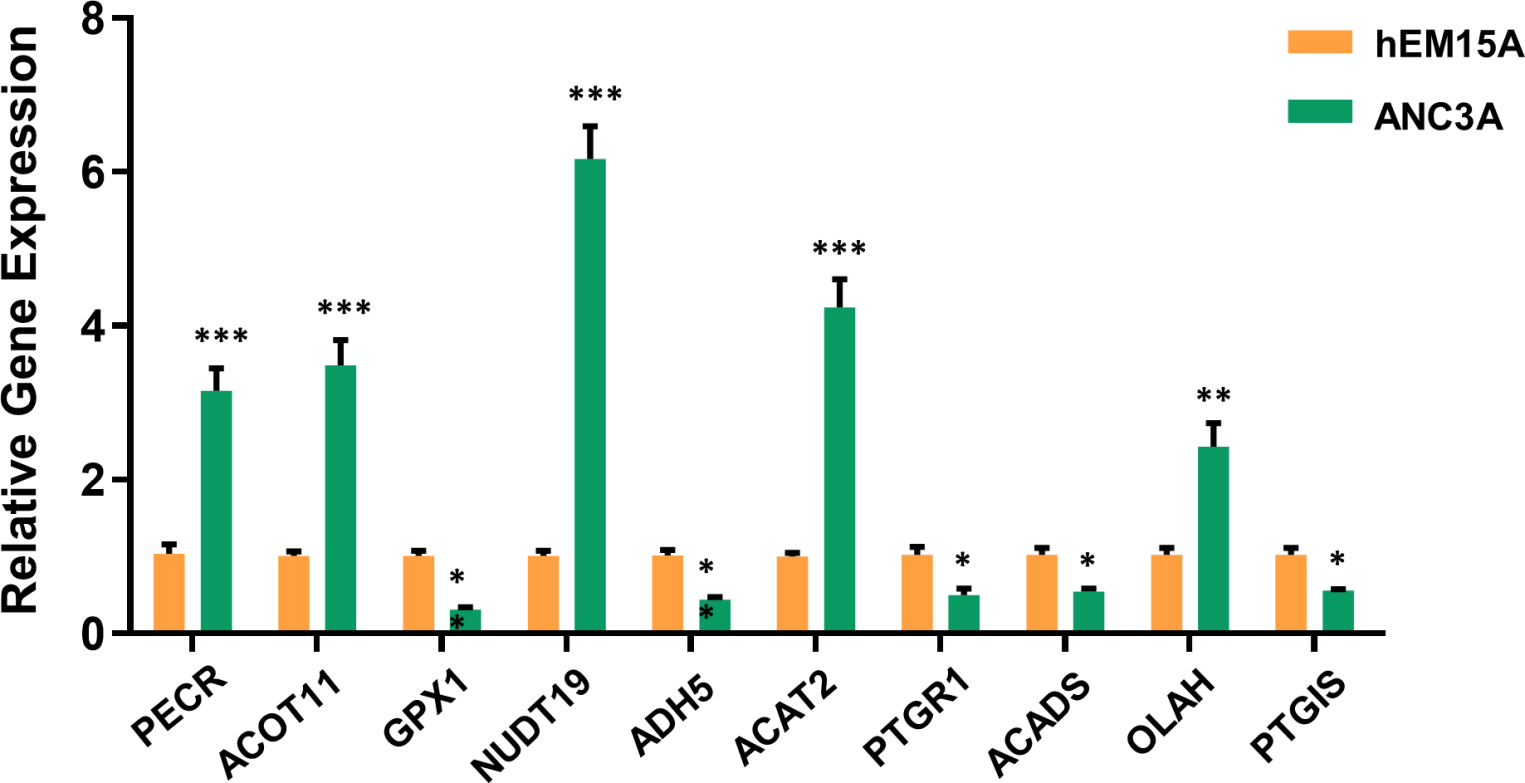
Validation of 10-FAMGs expression levels between UCEC cells and controls. ^∗^ *P*<0.05, ^∗∗^ *P*<0.01, and ^∗∗∗^ *P*<0.001. FAMGs: fatty acid metabolism-related genes. UCEC: Uterine corpus endometrial carcinoma.

To further validate the expression of FAMGs in this signature, we performed a qRT-PCR experiment to detect the difference between UCEC cells and normal endometrial cells. As expected, the expressions of PECR, OLAH, ACOT11, ACAT2, NUDT19 and PTGIS were upregulated, whereas GPX1, ADH5, PTGR1 and ACADS were downregulated (*P<* 0.05) in UCEC cells compared to normal cells (**Figure 8**). These were consistent with the above bioinformatic results.

## Discussion

UCEC is one of the most prevalently diagnosed gynecologic malignancy and ranks sixth among female tumors worldwide (22). In recent years, UCEC incidence and mortality have been increased with a younger trend. Early-stage UCEC could be surgically removed followed by chemoradiotherapy, with a 5-year survival rate of up to 90% (23). Nevertheless, advanced-stage patients at a high risk of recurrence had a worse prognosis, some prospective trials have attempted to identify these patients to develop effective adjuvant therapy (24–26), but to date, no interventions have been proven to improve OS. Traditional clinicopathological parameters are insufficient for precisely forecasting the outcome of UCEC, as patients in same clinical stage may exhibit distinct clinical features (27). As a result, the discovery of novel biomarkers for the prognosis and therapy of UCEC has become an urgent clinical issue to be resolved.

It is widely recognized that a major cause of the development of UCEC is obesity (28), which is linked to fatty acid oxidation, synthesis, accumulation and metabolic regulation. Additionally, a majority of FAMGs are known to be tightly related to malignancy and prognosis of cancers. The evidence supports a potent prognostic value of FAMGs for UCEC patients. The advent of bioinformatics has allowed us to examine the specific pattern of FAMGs for UCEC. In our study, a prognostic signature with 10 FAMGs was designed to predict UCEC patients’ survival time based on TCGA cohort. Among them, the expression levels of six genes (PECR, OLAH, ACOT11, ACAT2, NUDT19, PTGIS) were significantly upregulated and 4 genes (GPX1, ADH5, PTGR1, ACADS) were downregulated in UCEC samples compared to normal tissues, which have been validated by PCR experiment. Substantial evidence revealed that most of these FAMGs functioned as oncogenes or tumor-suppressor genes in various cancers (29–36). We also determined the prevalence of genetic mutation in UCEC samples, as well as CNV alterations. These confirmed the significant effect of fatty acid metabolism on UCEC malignant progression. Furthermore, a scoring system was created to identify and verify the prognostic value of FAMGs-based risk signature, allowing for the effective risk categorization of UCEC. High-FAMGs UCEC patients had a worse OS or PFS compared with low-FAMGs group. Importantly, FAMGs-based risk signature exhibited great accuracy and independence in predicting UCEC’ prognosis than other factors. In addition, a nomogram integrating our signature and patient characteristics was created as a superior tool to predict the survival of UCEC. We also discovered that this signature offered predictive value for different subgroup of patients with specific clinical characteristics. These findings indicated that FAMGs-based risk signature may be a reliable predictor of UCEC.

Tumor development is heavily reliant on the TME, which is complex milieu comprising cancer, stromal and immune cells, as well as microvessels and various chemicals (37). In TME, there are diverse regulatory mechanisms that promote immune tolerance and immune escape, in addition to loss of antigen presentation and upregulation of immune checkpoints, also including cellular metabolic reprogramming. Tumor metabolism not only modulates the signaling for tumorigenesis and survival, but also an antitumor immune response by releasing intermediate metabolites to affect the expression of immune molecules (38). FAM is a vital metabolic route involved in the immune response, which contains anabolic and catabolic activities for energy homeostasis, as well as metabolites generation that keeps cell membrane structure and function, stores energy and allows cross-talk between tumor and immune cells (39).

In our study, the effect of FAMGs in the risk signature on UCEC TME was further investigated. Patients with low- and high-FAMGs displayed remarkably different immune status. Following processing by ESTIMATE algorithm, it was discovered that low-FAMGs group had a higher estimate score relative to high-FAMGs group, indicating this risk signature may be able to function as a new immune indicator in UCEC. Then, TIDE technique was applied to anticipate clinical response to immune checkpoints and suggested that high-FAMGs patients were more effective to immunotherapy. We continue to investigate the TMB of UCEC patients. The results showed that patients with higher TMB possessed a better prognosis than those with lower TMB. Similar findings have been observed in other cancers (40–42), highlighting the possibility that TMB might serve as a prognostic marker for guiding more efficient immunotherapeutic approaches (43). By combing with FAMGs risk score, patients with low-TMB/high-FAMGs had the lowest survival probability than other groups. In addition, given the importance of HLA-related genes to immune system (44), we compared their expression levels in high- and low-FAMGs group, and found that most of these genes were generally increased in low-FAMGs group. Moreover, the resistance and sensitivity of common targeted drugs were measured to evaluate the prognostic ability of this risk model for therapeutic outcomes. These findings proved that FAMGs-based risk signature was associated with immune status and tumor treatment of UCEC.

Nevertheless, our study also has several limitations. First, the research was completely performed based on TCGA database, but lacked external cohorts for validation. Second, the regulatory mechanism of fatty acid metabolism in UCEC TIME warrants further investigation. Third, the value of FAMGs-based risk signature for clinical application requires multi-center, large sample trials to be processed.

In conclusion, we identified significant FAMGs in UCEC and constructed a FAMGs-based risk signature to predict the patients’ prognosis using systematic bioinformatic analyses. Patients with high-FAMGs had a worse prognosis than low-FAMGs patients. This signature might be regarded as an independent indicator to estimate the survival time, immune response and treatment effect. This study provided us a new understanding and direction on the evolution of FAMGs in UCEC.

## Data availability statement

The datasets presented in this study can be found in online repositories. The names of the repository/repositories and accession number(s) can be found in the article/**Supplementary Material**.

## Author contributions

CG, YH, LC, and YL designed this work. YW, YB, and NZ analyzed the data, FJ and HZ wrote this manuscript. LZ edited and revised the manuscript. All authors contributed to the article and approved the submitted version.

## Funding

This research was supported by Hainan Province Clinical Medical Center.

## Acknowledgments

The authors gratefully acknowledge the Cancer Genome Atlas (TCGA) database, which made the data available. The authors also thank Hainan Province Clinical Medical Center for research support.

## Conflict of interest

The authors declare that the research was conducted in the absence of any commercial or financial relationships that could be construed as a potential conflict of interest.

## Publisher’s note

All claims expressed in this article are solely those of the authors and do not necessarily represent those of their affiliated organizations, or those of the publisher, the editors and the reviewers. Any product that may be evaluated in this article, or claim that may be made by its manufacturer, is not guaranteed or endorsed by the publisher.

## References

[1] Siegel RL Miller KD Fuchs HE Jemal A . Cancer statistics, 2022. CA Cancer J Clin (2022) 72:7–33. doi: 10.3322/caac.21708 35020204

[2] Xia C Dong X Li H Cao M Sun D He S . Cancer statistics in China and united states, 2022: profiles, trends, and determinants. Chin Med J (Engl) (2022) 135:584–90. doi: 10.1097/CM9.0000000000002108 PMC8920425 35143424

[3] Lu KH Broaddus RR . Endometrial cancer. N Engl J Med (2020) 383:2053–64. doi: 10.1056/NEJMra1514010 33207095

[4] Lee YY Choi MC Park JY Suh DH Kim JW . Major clinical research advances in gynecologic cancer in 2020. J Gynecol Oncol (2021) 32:e53. doi: 10.3802/jgo.2021.32.e53 34085794 PMC8192228

[5] Hinshaw DC Shevde LA . The tumor microenvironment innately modulates cancer progression. Cancer Res (2019) 79:4557–66. doi: 10.1158/0008-5472.CAN-18-3962 PMC6744958 31350295

[6] Xia L Oyang L Lin J Tan S Han Y Wu N . The cancer metabolic reprogramming and immune response. Mol Cancer (2021) 20:28. doi: 10.1186/s12943-021-01316-8 33546704 PMC7863491

[7] Munir R Lisec J Swinnen JV Zaidi N . Too complex to fail? targeting fatty acid metabolism for cancer therapy. Prog Lipid Res (2022) 85:101143. doi: 10.1016/j.plipres.2021.101143 34856213

[8] Qi Y Chen D Lu Q Yao Y Ji C . Bioinformatic profiling identifies a fatty acid metabolism-related gene risk signature for malignancy, prognosis, and immune phenotype of glioma. Dis Markers (2019) 2019:3917040. doi: 10.1155/2019/3917040 31885736 PMC6914924

[9] Ding C Shan Z Li M Chen H Li X Jin Z . Characterization of the fatty acid metabolism in colorectal cancer to guide clinical therapy. Mol Ther Oncolytics (2021) 20:532–44. doi: 10.1016/j.omto.2021.02.010 PMC7941088 33738339

[10] Tang Y Tian W Xie J Zou Y Wang Z Li N . Prognosis and dissection of immunosuppressive microenvironment in breast cancer based on fatty acid metabolism-related signature. Front Immunol (2022) 13:843515. doi: 10.3389/fimmu.2022.843515 35432381 PMC9009264

[11] Jiang F Luo F Zeng N Mao Y Tang X Wang J . Characterization of fatty acid metabolism-related genes landscape for predicting prognosis and aiding immunotherapy in glioma patients. Front Immunol (2022) 13:902143. doi: 10.3389/fimmu.2022.902143 35903107 PMC9315048

[12] Lu L Hu Y Wang C Jiang F Wu C . Methylation and expression of the exercise-related TLR1 gene is associated with low grade glioma prognosis and outcome. Front Mol Biosci (2021) 8:747933. doi: 10.3389/fmolb.2021.747933 34869584 PMC8635206

[13] Jiang F Hu Y Liu X Wang M Wu C . Methylation pattern mediated by m(6)A regulator and tumor microenvironment invasion in lung adenocarcinoma. Oxid Med Cell Longev (2022) 2022:2930310. doi: 10.1155/2022/2930310 35035657 PMC8756160

[14] Zhang S Chang W Wu H Wang YH Gong YW Zhao YL . Pan-cancer analysis of iron metabolic landscape across the cancer genome atlas. J Cell Physiol (2020) 235:1013–24. doi: 10.1002/jcp.29017 31240715

[15] Ritchie ME Phipson B Wu D Hu Y Law CW Shi W . Limma powers differential expression analyses for RNA-sequencing and microarray studies. Nucleic Acids Res (2015) 43:e47. doi: 10.1093/nar/gkv007 25605792 PMC4402510

[16] Engebretsen S Bohlin J . Statistical predictions with glmnet. Clin Epigenet (2019) 11:123. doi: 10.1186/s13148-019-0730-1 PMC6708235 31443682

[17] Mandrekar JN . Receiver operating characteristic curve in diagnostic test assessment. J Thorac Oncol (2010) 5:1315–6. doi: 10.1097/JTO.0b013e3181ec173d 20736804

[18] Pomaznoy M Ha B Peters B . GOnet: a tool for interactive gene ontology analysis. BMC Bioinf (2018) 19:470. doi: 10.1186/s12859-018-2533-3 PMC6286514 30526489

[19] Kanehisa M Furumichi M Tanabe M Sato Y Morishima K . KEGG: new perspectives on genomes, pathways, diseases and drugs. Nucleic Acids Res (2017) 45:D353–353D361. doi: 10.1093/nar/gkw1092 27899662 PMC5210567

[20] Park SY . Nomogram: An analogue tool to deliver digital knowledge. J Thorac Cardiovasc Surg (2018) 155:1793. doi: 10.1016/j.jtcvs.2017.12.107 29370910

[21] Geeleher P Cox N Huang RS . pRRophetic: an r package for prediction of clinical chemotherapeutic response from tumor gene expression levels. PloS One (2014) 9:e107468. doi: 10.1371/journal.pone.0107468 25229481 PMC4167990

[22] Sung H Ferlay J Siegel RL Laversanne M Soerjomataram I Jemal A . Global cancer statistics 2020: GLOBOCAN estimates of incidence and mortality worldwide for 36 cancers in 185 countries. CA Cancer J Clin (2021) 71:209–49. doi: 10.3322/caac.21660 33538338

[23] McEachron J Marshall L Zhou N Tran V Kanis MJ Gorelick C . Evaluation of survival, recurrence patterns and adjuvant therapy in surgically staged high-grade endometrial cancer with retroperitoneal metastases. Cancers (Basel) (2021) 13:2052. doi: 10.3390/cancers13092052 PMC8123054 33922792

[24] Matei D Filiaci V Randall ME Mutch D Steinhoff MM DiSilvestro PA . Adjuvant chemotherapy plus radiation for locally advanced endometrial cancer. N Engl J Med (2019) 380:2317–26. doi: 10.1056/NEJMoa1813181 PMC6948006 31189035

[25] Randall ME Filiaci V McMeekin DS von Gruenigen V Huang H Yashar CM . Phase III trial: Adjuvant pelvic radiation therapy versus vaginal brachytherapy plus Paclitaxel/Carboplatin in high-intermediate and high-risk early stage endometrial cancer. J Clin Oncol (2019) 37:1810–8. doi: 10.1200/JCO.18.01575 PMC6804858 30995174

[26] van den Heerik A Horeweg N de Boer SM Bosse T Creutzberg CL . Adjuvant therapy for endometrial cancer in the era of molecular classification: radiotherapy, chemoradiation and novel targets for therapy. Int J Gynecol Cancer (2021) 31:594–604. doi: 10.1136/ijgc-2020-001822 33082238 PMC8020082

[27] Zhou C Li C Yan F Zheng Y . Identification of an immune gene signature for predicting the prognosis of patients with uterine corpus endometrial carcinoma. Cancer Cell Int (2020) 20:541. doi: 10.1186/s12935-020-01560-w 33292199 PMC7650210

[28] Hoy AJ Nagarajan SR Butler LM . Tumour fatty acid metabolism in the context of therapy resistance and obesity. Nat Rev Cancer (2021) 21:753–66. doi: 10.1038/s41568-021-00388-4 34417571

[29] Liang C Wang X Zhang Z Xiao F Feng H Ma Q . ACOT11 promotes cell proliferation, migration and invasion in lung adenocarcinoma. Transl Lung Cancer Res (2020) 9:1885–903. doi: 10.21037/tlcr-19-509 PMC7653140 33209610

[30] Weng M Zhang H Hou W Sun Z Zhong J Miao C . ACAT2 promotes cell proliferation and associates with malignant progression in colorectal cancer. Onco Targets Ther (2020) 13:3477–88. doi: 10.2147/OTT.S238973 PMC7187938 32425549

[31] Lan C Wang Y Su X Lu J Ma S . LncRNA LINC00958 activates mTORC1/P70S6K signalling pathway to promote epithelial-mesenchymal transition process in the hepatocellular carcinoma. Cancer Invest (2021) 39:539–49. doi: 10.1080/07357907.2021.1929282 33979257

[32] Dai D Chen B Feng Y Wang W Jiang Y Huang H . Prognostic value of prostaglandin I2 synthase and its correlation with tumor-infiltrating immune cells in lung cancer, ovarian cancer, and gastric cancer. Aging (Albany NY) (2020) 12:9658–85. doi: 10.18632/aging.103235 PMC7288932 32463792

[33] Zhao Y Wang H Zhou J Shao Q . Glutathione peroxidase GPX1 and its dichotomous roles in cancer. Cancers (Basel) (2022) 14:2560. doi: 10.3390/cancers14102560 PMC9139801 35626163

[34] Li N Li N Wen S Li B Zhang Y Liu Q . HSP60 regulates lipid metabolism in human ovarian cancer. Oxid Med Cell Longev (2021) 2021:6610529. doi: 10.1155/2021/6610529 34557266 PMC8452972

[35] Xue L Zhu Z Wang Z Li H Zhang P Wang Z . Knockdown of prostaglandin reductase 1 (PTGR1) suppresses prostate cancer cell proliferation by inducing cell cycle arrest and apoptosis. Biosci Trends (2016) 10:133–9. doi: 10.5582/bst.2016.01045 27150108

[36] Wu Q Yan T Chen Y Chang J Jiang Y Zhu D . Integrated analysis of expression and prognostic values of acyl-CoA dehydrogenase short-chain in colorectal cancer. Int J Med Sci (2021) 18:3631–43. doi: 10.7150/ijms.63953 PMC8579304 34790035

[37] Anderson NM Simon MC . The tumor microenvironment. Curr Biol (2020) 30:R921–921R925. doi: 10.1016/j.cub.2020.06.081 32810447 PMC8194051

[38] Martínez-Reyes I Chandel NS . Cancer metabolism: looking forward. Nat Rev Cancer (2021) 21:669–80. doi: 10.1038/s41568-021-00378-6 34272515

[39] Wang Y Wang Y Ren Y Zhang Q Yi P Cheng C . Metabolic modulation of immune checkpoints and novel therapeutic strategies in cancer. Semin Cancer Biol (2022) 86:542–65. doi: 10.1016/j.semcancer.2022.02.010 35151845

[40] Guan X Xu ZY Chen R Qin JJ Cheng XD . Identification of an immune gene-associated prognostic signature and its association with a poor prognosis in gastric cancer patients. Front Oncol (2020) 10:629909. doi: 10.3389/fonc.2020.629909 33628738 PMC7898907

[41] Li P Hao S Ye Y Wei J Tang Y Tan L . Identification of an immune-related risk signature correlates with immunophenotype and predicts anti-PD-L1 efficacy of urothelial cancer. Front Cell Dev Biol (2021) 9:646982. doi: 10.3389/fcell.2021.646982 33816497 PMC8012532

[42] Pei JP Zhang CD Yusupu M Zhang C Dai DQ . Screening and validation of the hypoxia-related signature of evaluating tumor immune microenvironment and predicting prognosis in gastric cancer. Front Immunol (2021) 12:705511. doi: 10.3389/fimmu.2021.705511 34249015 PMC8267919

[43] Romero D . TMB is linked with prognosis. Nat Rev Clin Oncol (2019) 16:336. doi: 10.1038/s41571-019-0206-4 30932077

[44] Paulson KG Voillet V McAfee MS Hunter DS Wagener FD Perdicchio M . Acquired cancer resistance to combination immunotherapy from transcriptional loss of class I HLA. Nat Commun (2018) 9:3868. doi: 10.1038/s41467-018-06300-3 30250229 PMC6155241

