# Supplementary figures and images for: Integrated bioinformatics analysis and experimental validation reveals fatty acid metabolism-related prognostic signature and immune responses for uterine corpus endometrial carcinoma
Source: Front Oncol. 2022 Nov 9;12:1030246. doi: 10.3389/fonc.2022.1030246 (PMC9682070; doi:10.3389/fonc.2022.1030246)

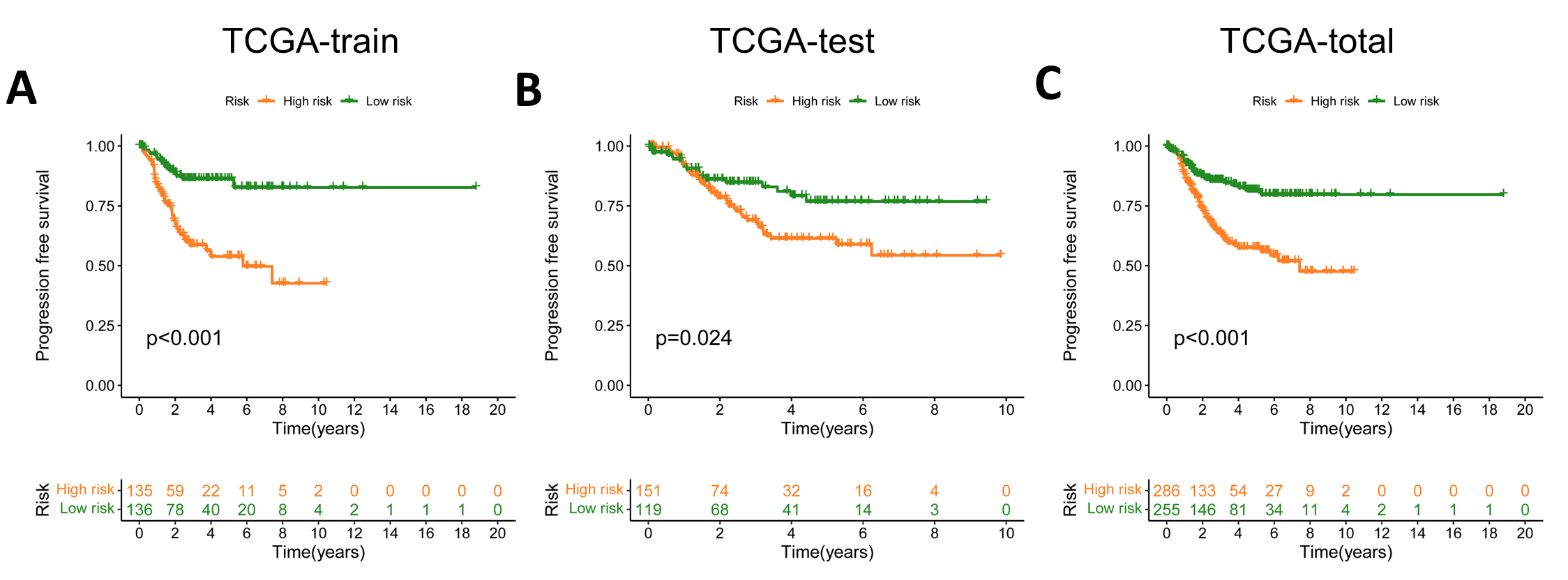

Supplement: Supplementary file 1 [file Image_1.tif]

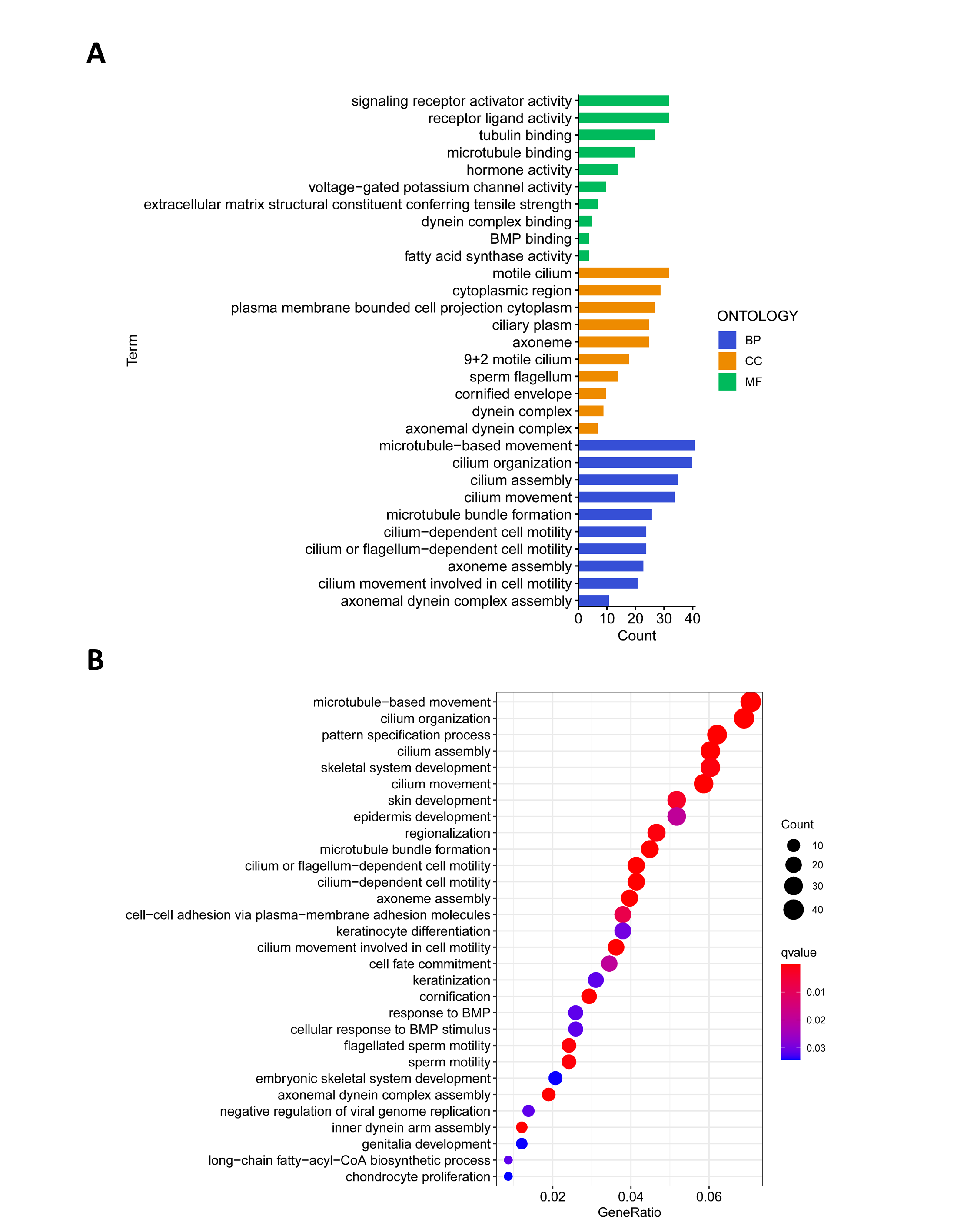

Supplement: Supplementary file 2 [file Image_2.tif]

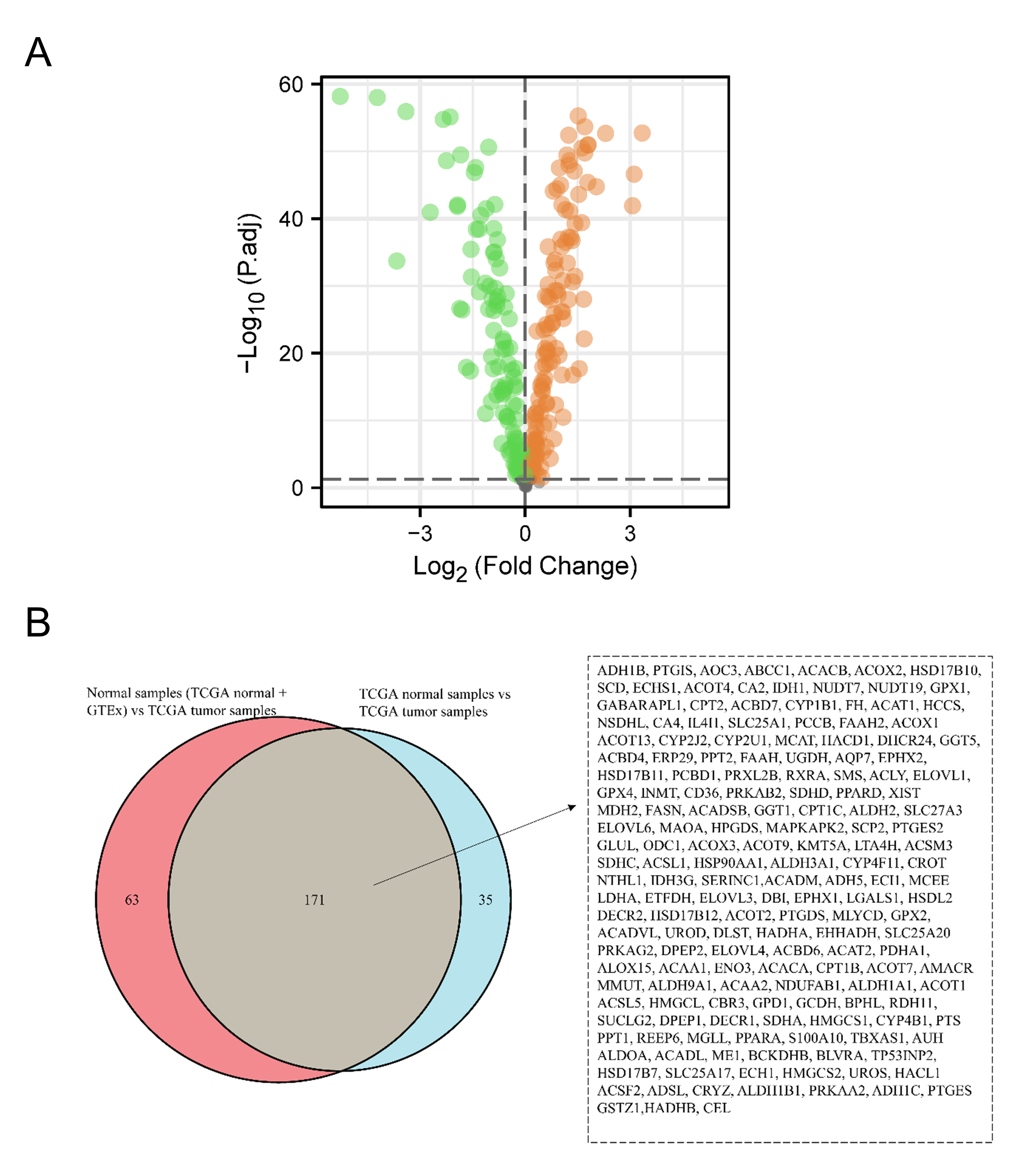

Supplement: Supplementary file 3 [file Image_3.tif]
