# Supplementary material for: Integrated bioinformatics analysis and experimental validation reveals fatty acid metabolism-related prognostic signature and immune responses for uterine corpus endometrial carcinoma
Source: Front Oncol. 2022 Nov 9;12:1030246. doi: 10.3389/fonc.2022.1030246 (PMC9682070; doi:10.3389/fonc.2022.1030246)
Supplement: Supplementary file 4 [file Table_1.docx]

**Table S1**

**Primer sequences used in this study**

| **Primer set** | **Primers** | **Sequence (5’-3’)** | **Product size (bp)** |
| --- | --- | --- | --- |
| PECR | F | 5'- AGTCATTCCCATACAATGCAACA -3' | 155 |
|  | R | 5'- GCCATCCCTTAGAACTGATGTG -3' |  |
| ACOT11 | F | 5'- CATCGTGAACAATGCCTTCAAAC -3' | 124 |
|  | R | 5'- GTCCAGGACCACAAAGGTCAT -3' |  |
| GPX1 | F | 5'- CAGTCGGTGTATGCCTTCTCG -3' | 105 |
|  | R | 5'- GAGGGACGCCACATTCTCG -3' |  |
| NUDT19 | F | 5'- CCCCACAGTTCTACGAAGTGA -3' | 85 |
|  | R | 5'- TCTAATGCACGACCCAAACAAA -3' |  |
| ADH5 | F | 5'- ATGGCGAACGAGGTTATCAAG -3' | 202 |
|  | R | 5'- CATGTCCCAAGATCACTGGAAAA -3' |  |
| ACAT2 | F | 5'- GCGGACCATCATAGGTTCCTT -3' | 181 |
|  | R | 5'- ACTGGCTTGTCTAACAGGATTCT -3' |  |
| PTGR1 | F | 5'- GACAACGCACTCCATTTCTGA -3' | 178 |
|  | R | 5'- TGCTGCATTAACCATCACTGTT -3' |  |
| ACADS | F | 5'- CGGCAGTTACACACCATCTAC -3' | 104 |
|  | R | 5'- GCAATGGGAAACAACTCCTTCTC -3' |  |
| OLAH | F | 5'- CCTGGCATCGCATTCCCAA -3' | 175 |
|  | R | 5'- GGTACGTTAGAGGTGCAACTTC -3' |  |
| PTGIS | F | 5'- CTGGTTGGGGTATGCCTTGG -3' | 246 |
|  | R | 5'- TCATCACTGGGGCTGTAATGT -3' |  |
